# Real-world outcomes of faricimab in treatment-naïve patients with neovascular age-related macular degeneration

**DOI:** 10.1186/s12886-026-05019-w

**Published:** 2026-06-11

**Authors:** Woohyok Chang, Jinhee Kim, Junsik Jeon, Soyeon Park

**Affiliations:** Chang’s Retina Center, 290-1 Daemyeong-ro, Nam-gu, Daegu, South Korea

**Keywords:** Asian, Faricimab, Neovascular age-related macular degeneration, Polypoidal choroidal vasculopathy, Real-world, Treatment-naïve

## Abstract

**Background:**

To evaluate real-world efficacy, durability, and safety of faricimab in treatment-naïve Asian patients with neovascular age-related macular degeneration (nAMD).

**Methods:**

Retrospective study of treatment-naïve nAMD eyes treated with faricimab at a single centre in South Korea. After four monthly loading doses, treatment intervals (Q8W, Q12W, Q16W) were assigned based on disease activity at weeks 20 and 24. Comprehensive assessments included best-corrected visual acuity (BCVA), central subfield thickness (CST), pigment epithelial detachment (PED) height, subfoveal choroidal thickness (SFCT), and intraretinal/subretinal fluid (IRF/SRF) presence.

**Results:**

Eighty-eight treatment-naïve eyes were included. Mean age was 72.8 (9.0) years. At week 20, BCVA improved from 62.1 (15.4) to 69.3 (14.6) ETDRS letters (+ 7.1 letters, *P* < 0.001). CST decreased from 414 (150) to 280 (94) µm (− 134 μm, *P* < 0.001). PED height decreased from 297 (167) to 171 (116) µm (− 43%, *P* < 0.001). SFCT decreased from 208 (91) to 184 (83) µm (− 12%, *P* < 0.001), with PCV eyes showing the greatest reduction (− 32 μm, *P* < 0.001). IRF presence (1 mm/6 mm) decreased from 33%/34% to 7%/9%, and SRF from 78%/84% to 12%/20% at week 20. At week 60, 52.3% achieved Q16W and 67.0% achieved ≥Q12W intervals. Among 168 eyes in the safety analysis, there was 1 case of mild vitritis and 2 cases of RPE tear, with no cases of endophthalmitis or retinal vasculitis reported.

**Conclusions:**

Initiation of faricimab in treatment-naïve nAMD patients resulted in favourable visual and anatomical outcomes with durable treatment intervals and a low incidence of adverse events in a real-world setting.

## Background

Neovascular age-related macular degeneration (nAMD) is a leading cause of central vision loss in the elderly [1]. Anti-vascular endothelial growth factor (anti-VEGF) therapy has become the standard treatment, yet real-world outcomes often fall short of clinical trial results due to the high treatment burden and subsequent undertreatment [2–4]. 

Faricimab is the first bispecific antibody designed for intraocular use that independently binds and neutralizes both angiopoietin-2 (Ang-2) and VEGF-A [5]. By targeting Ang-2, faricimab addresses vascular instability and inflammation that VEGF inhibition alone cannot control [6, 7]. The phase 3 TENAYA and LUCERNE trials demonstrated non-inferior visual outcomes compared with aflibercept while enabling extended dosing intervals up to every 16 weeks (Q16W) in approximately 45% of patients at week 48, increasing to 59–67% at week 112 [8, 9]. 

The TENAYA/LUCERNE Asian country subgroup analysis by Takahashi et al. showed remarkable durability outcomes in 61 faricimab-treated patients: Q16W achievement of 59.6% and ≥Q12W of 91.2% at week 48, substantially exceeding non-Asian results [10]. Post hoc analyses demonstrated that faricimab achieved superior anatomical outcomes during the head-to-head dosing phase, with greater reductions in CST, PED height, and faster fluid resolution compared with aflibercept [11–13].

Asian patients with nAMD exhibit distinct characteristics, including higher prevalence of polypoidal choroidal vasculopathy (PCV), accounting for 22–62% of cases [14, 15]. Eyes with PCV demonstrate elevated Ang-2 levels, suggesting that dual Ang-2/VEGF-A inhibition may be particularly beneficial in this population [16]. Pachychoroid spectrum diseases, common in Asian populations, may also respond favorably to Ang-2 inhibition [17]. 

While the Asian subgroup of pivotal trials demonstrated excellent outcomes, real-world validation in treatment-naïve Asian patients with comprehensive anatomical endpoints remains limited. This study aimed to evaluate the efficacy, durability, and safety of faricimab in treatment-naïve Asian nAMD patients using a treatment regimen adapted from the pivotal trials in a real-world clinical setting.

## Methods

### Study design and participants

This retrospective study was conducted at Chang’s Retina Center, Daegu, South Korea. The study adhered to the tenets of the Declaration of Helsinki and was granted an exemption from full ethical review by the Public Institutional Review Board (Public IRB) designated by the Ministry of Health and Welfare of South Korea and operated by the Korea National Institute for Bioethics Policy (P01-202510-01-057). The requirement for informed consent was waived due to the retrospective nature of the study using de-identified medical records. We reviewed medical records of all patients who initiated faricimab treatment between October 2023 and July 2024.

Inclusion criteria were: diagnosis of nAMD with subfoveal or juxtafoveal MNV confirmed by multimodal imaging; treatment-naïve status with no prior anti-VEGF therapy; completion of four monthly loading injections; and follow-up through 1 year. Exclusion criteria were: prior anti-VEGF or photodynamic therapy; switching to other anti-VEGF agents during follow-up (mandated under Korean reimbursement criteria for eyes not meeting response thresholds, as detailed in the patient disposition); and incomplete imaging data.

### Treatment protocol

All patients received four loading doses of faricimab 6.0 mg at 4-week intervals. Treatment intervals were determined based on disease activity assessed at weeks 20 and 24. Active disease was defined as: new or increased IRF or SRF within the central 6 mm zone deemed clinically significant by the investigator; increase in PED height; new macular hemorrhage; or BCVA decrease ≥ 15 letters from baseline. Patients with activity at week 20 received Q8W dosing; those inactive at week 20 but active at week 24 received Q12W dosing; those inactive at both visits received Q16W dosing. This represented a one-time interval assignment rather than a continuous treat-and-extend regimen; the assigned interval was maintained through week 60. The minimum treatment interval permitted was Q8W. No eye in the included cohort developed exacerbation severe enough to require shortening of the assigned interval during follow-up.

### Outcome measures

Primary outcomes were BCVA and CST (µm) changes from baseline to week 20. BCVA was measured using decimal visual acuity charts and converted to ETDRS letter equivalents for analysis. PED was defined as present when the maximum PED height exceeded 100 μm; PED width was not incorporated into the definition. Maximum PED height was measured manually using the built-in caliper tool of the OCT review software at the point of maximum elevation within the 6 × 6 mm macular area at baseline, with this location tracked manually throughout follow-up; no automated segmentation software was used. SFCT was measured manually with the same caliper tool as the vertical distance from the outer border of the retinal pigment epithelium to the chorioscleral interface beneath the fovea. Secondary anatomical outcomes included changes in maximum PED height (among eyes with baseline PED > 100 μm), changes in SFCT, and changes in IRF/SRF presence in the central 1 mm and 6 mm zones. Secondary efficacy outcomes included treatment interval distribution at week 60.

Safety was assessed in all 168 eyes receiving ≥ 1 injection. Clinically significant intraocular inflammation (IOI) was defined as anterior chamber cells > 1+.

### MNV classification

MNV subtypes were classified as type 1, type 2, type 3, or PCV based on multimodal imaging including spectral-domain optical coherence tomography (SD-OCT; Spectralis, Heidelberg Engineering, Heidelberg, Germany), fluorescein angiography, and indocyanine green angiography.

### Statistical analysis

Statistical analyses were performed using R software (version 4.3.3; R Foundation for Statistical Computing, Vienna, Austria). Continuous variables were expressed as mean (standard deviation [SD]). Normality was assessed using the Shapiro-Wilk test. Changes from baseline in BCVA, CST, PED height, and SFCT were analysed using paired t-tests for normally distributed data or Wilcoxon signed-rank tests otherwise. Changes in fluid presence (IRF/SRF) over time, and zonal comparisons of fluid presence between the central 1 mm and 6 mm zones at each timepoint, were analysed using the McNemar test for paired categorical data. Treatment interval distribution was summarised descriptively, and the association between MNV subtype and Q16W achievement was assessed using the chi-square test. Data at weeks 28 and 44 were available only for patients with scheduled visits at those timepoints (Q8W and Q16W groups; *n* = 75), as Q12W patients did not have protocol-specified visits at these intervals. Statistical significance was set at a nominal *P* < 0.05.

## Results

### Patient disposition

Of 168 treatment-naïve eyes assessed for eligibility (176 eyes from 168 patients; the first-treated eye was selected for 8 bilateral cases), 80 were excluded for the following reasons: prior anti-VEGF therapy (*n* = 50); switched to alternative anti-VEGF agents under Korean reimbursement criteria (which require anatomical response after 3 loading injections and VA > 0.1 after the loading phase for continued faricimab coverage): VA ≤ 0.1 after 4 injections including 2 RPE tears (*n* = 10) and insufficient anatomical response after 3 injections (*n* = 5), totalling 15 eyes switched; lost to follow-up (*n* = 13); vitritis (*n* = 1); and other medical reasons (*n* = 1). Finally, 88 eyes completed the 1-year study protocol and were included in the efficacy analysis. Safety analysis included all 168 eyes.

### Baseline characteristics

Mean age was 72.8 (9.0) years; 53 (60.2%) were male. MNV subtypes included PCV (36.4%), type 1 (28.4%), type 2 (20.5%), and type 3 (14.8%). Mean baseline BCVA was 62.1 (15.4) ETDRS letters (approximately 20/63 Snellen equivalent); mean baseline CST was 414 (150) µm (Table 1).

**Table 1:** Baseline characteristics

Characteristic	Value
Age, years (mean ± SD)	72.8 ± 9.0
Sex, male (%)	53 (60.2%)
Eye, right (%)	37 (42.0%)
MNV subtype	
Type 1	25 (28.4%)
Type 2	18 (20.5%)
Type 3	13 (14.8%)
PCV	32 (36.4%)
BCVA, ETDRS letters (mean ± SD)	62.1 ± 15.4
CST, µm (mean ± SD)	414 ± 150
PED height, µm (mean ± SD)*	297 ± 167
SFCT, µm (mean ± SD)	208 ± 91
IRF presence (%)	33%
SRF presence (%)	78%

* Among eyes with baseline PED ≥ 100 μm (*n* = 72)

BCVA = best-corrected visual acuity; CST = central subfield thickness; ETDRS = Early Treatment Diabetic Retinopathy Study; IRF = intraretinal fluid; MNV = macular neovascularization; PCV = polypoidal choroidal vasculopathy; PED = pigment epithelial detachment; SD = standard deviation; SFCT = subfoveal choroidal thickness; SRF = subretinal fluid

### Visual acuity outcomes

**Fig. 1:**
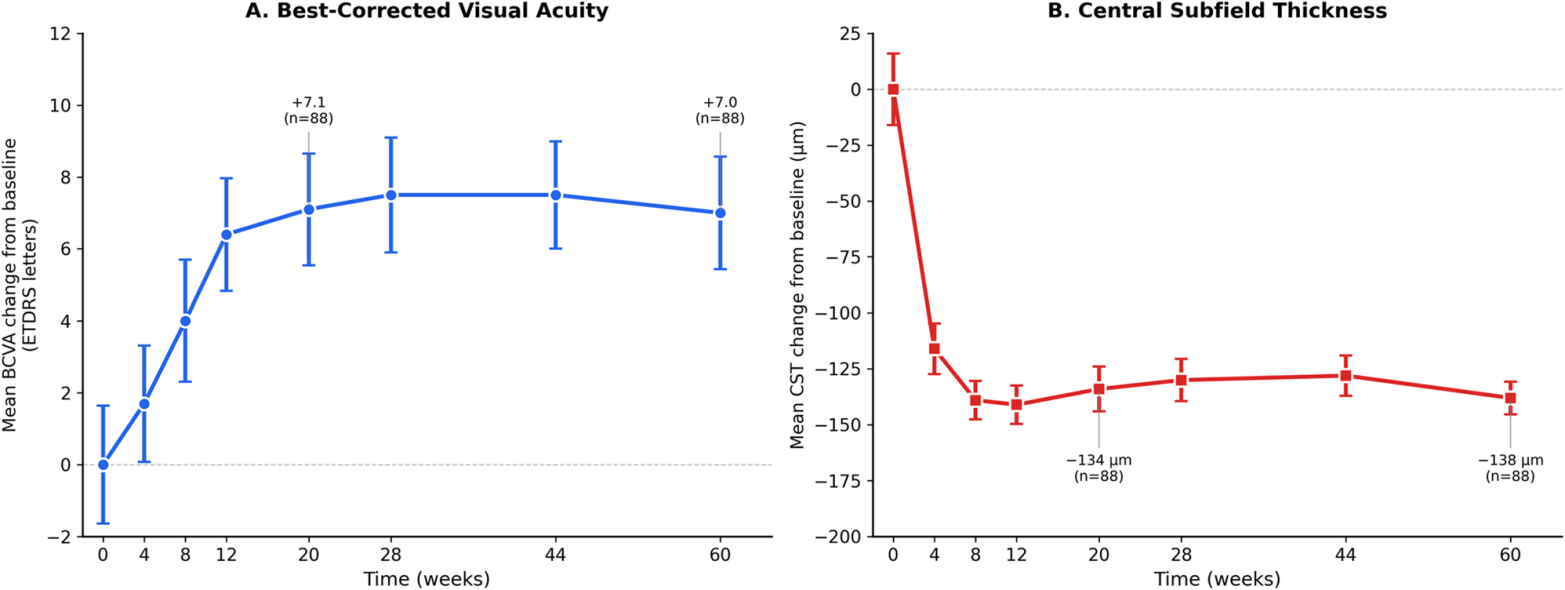
Mean change in BCVA and central subfield thickness from baseline. Mean change from baseline in (**A**) BCVA (ETDRS letters) and (**B**) CST (µm) through week 60. Error bars represent standard error of the mean (SEM). Values and sample sizes are shown at weeks 20 and 60. Data at weeks 28 (*n* = 66) and 44 (*n* = 75) include only patients with scheduled visits (Q8W and Q16W groups)

At week 20, mean BCVA improved to 69.3 (14.6) ETDRS letters (approximately 20/40 Snellen equivalent; mean logMAR 0.31), representing a gain of + 7.1 (11.7) letters (*P* < 0.001). Mean baseline BCVA corresponded to logMAR 0.46 (decimal Snellen 0.43), improving to logMAR 0.31 (decimal 0.58) at week 20 and logMAR 0.32 (decimal 0.58) at week 60. Visual gains were sustained throughout extended follow-up: +6.7 letters at week 44 (*n* = 75) and + 7.0 letters at week 60 (*n* = 88, 100% follow-up rate). At week 20, 52.4% achieved ≥ 5-letter (1-line) improvement and 26.2% achieved ≥ 10-letter (2-line) improvement (Table 2; Fig. 1A).

**Table 2:** Visual and anatomical outcomes

Parameter	Baseline (*n* = 88)	Week 20 (*n* = 88)	Week 28 (*n* = 66*)	Week 44 (*n* = 75*)	Week 60 (*n* = 88)
BCVA, ETDRS letters	62.1 ± 15.4	69.3 ± 14.6 (+ 7.1)	68.4 ± 15.1 (+ 5.5)	69.0 ± 14.1 (+ 6.4)	69.2 ± 14.7 (+ 7.0)
CST, µm	414 ± 150	280 ± 94 (− 134)	281 ± 88 (− 125)	285 ± 88 (− 121)	276 ± 69 (− 138)
PED height, µm†	297 ± 167	171 ± 116 (− 43%)	188 ± 116 (− 34%)	181 ± 114 (− 36%)	180 ± 107 (− 39%)
SFCT, µm	208 ± 91	184 ± 83 (− 12%)	185 ± 85 (− 7%)	187 ± 90 (− 10%)	176 ± 78 (− 15%)
IRF presence (1 mm/6 mm), %	33/34	7/9	14/15	10/11	3/3
SRF presence (1 mm/6 mm), %	78/84	12/20	17/26	19/30	21/27

Values are mean ± SD (change from baseline). All *P* < 0.001 vs. baseline

* Weeks 28 and 44 data include only patients with scheduled visits (Q8W and Q16W groups)

† Among eyes with baseline PED ≥ 100 μm; n varies: W20 *n* = 72, W28 *n* = 53, W44 *n* = 60, W60 *n* = 72

BCVA = best-corrected visual acuity; CST = central subfield thickness; ETDRS = Early Treatment Diabetic Retinopathy Study; IRF = intraretinal fluid; PED = pigment epithelial detachment; SD = standard deviation; SFCT = subfoveal choroidal thickness; SRF = subretinal fluid

### Central subfield thickness

CST decreased significantly from 414 (150) µm at baseline to 280 (94) µm at week 20 (− 134 (134) µm, *P* < 0.001). Anatomical improvements remained stable through week 44 (284 (87) µm, *n* = 75) and week 60 (276 (69) µm, *n* = 88), demonstrating durable disease control over 1 year of follow-up (Fig. 1B).

### Pigment epithelial detachment outcomes

**Fig. 2:**
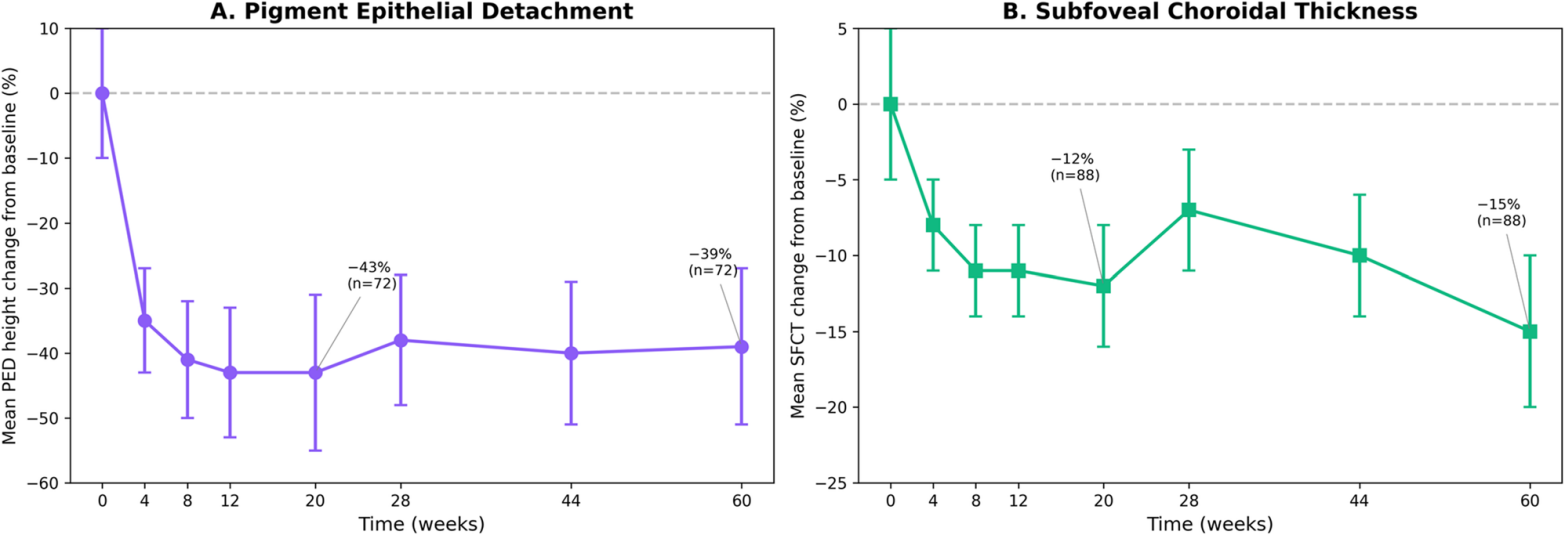
Mean change in PED height and subfoveal choroidal thickness (SFCT) from Baseline. Mean percentage change from baseline in (**A**) PED height among eyes with baseline PED > 100 μm and (**B**) SFCT through week 60. Error bars represent standard error of the mean (SEM). Values and sample sizes are shown at weeks 20 and 60. Data at weeks 28 and 44 include only patients with scheduled visits (Q8W and Q16W groups); PED: *n* = 53 and *n* = 60; SFCT: *n* = 66 and *n* = 75, respectively

Among eyes with baseline PED height > 100 μm (*n* = 72), mean maximum PED height decreased from 297 (167) µm at baseline to 171 (116) µm at week 20 (− 126 μm, − 43%, *P* < 0.001). This reduction was maintained through week 44 (179 (112) µm, *n* = 65) and week 60 (180 (107) µm, *n* = 72), demonstrating durable anatomical improvement (Fig. 2A).

### Subfoveal choroidal thickness

SFCT decreased from 208 (91) µm at baseline to 184 μm at week 20 (− 24 μm, − 12%, *P* < 0.001) and 176 μm at week 60 (− 32 μm, − 15%, *P* < 0.001), demonstrating sustained choroidal thinning over the study period (*n* = 88) (Fig. 2B). SFCT reduction was greatest in PCV eyes (−43 μm, −17%, *P* < 0.001), consistent with the pachychoroid phenotype, followed by type 1 (− 29 μm, − 14%), type 2 (− 31 μm, − 17%), and type 3 MNV (− 12 μm, − 9%).

### Intraretinal and subretinal fluid resolution

**Fig. 3:**
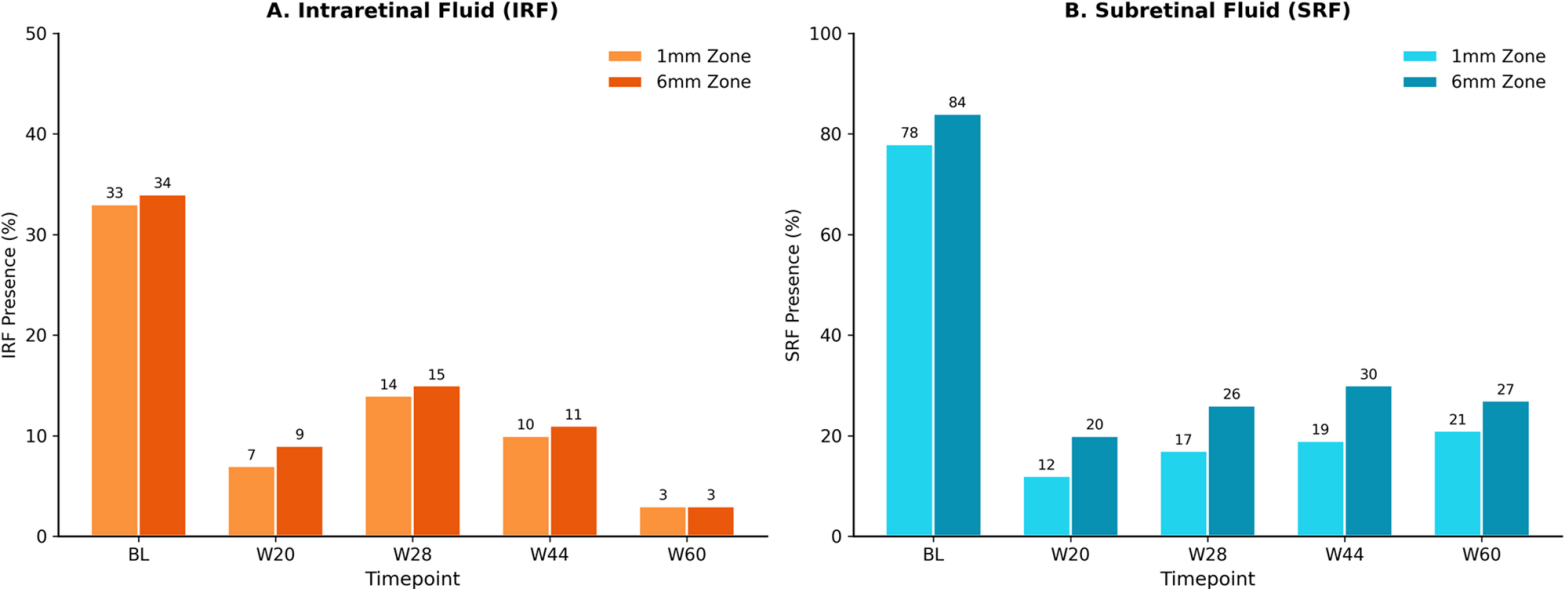
Intraretinal and subretinal fluid presence over time. Percentage of eyes with intraretinal fluid (IRF) and subretinal fluid (SRF) at each timepoint through week 60

IRF presence (1 mm/6 mm) decreased from 33%/34% at baseline to 7%/9% at week 20 and 3%/3% at week 60. SRF presence (1 mm/6 mm) decreased from 78%/84% at baseline to 12%/20% at week 20; however, SRF presence increased to 21%/27% at week 60 (Fig. 3). At week 20, SRF presence was significantly higher in the 6 mm zone than in the central 1 mm zone (19.5% vs. 12.2%; McNemar exact *P* = 0.031), whereas the difference was not significant at baseline (*P* = 0.125) or week 60 (*P* = 0.625). For IRF, no significant zonal difference was observed at any timepoint (all *P* > 0.99).

### Treatment interval distribution

**Fig. 4:**
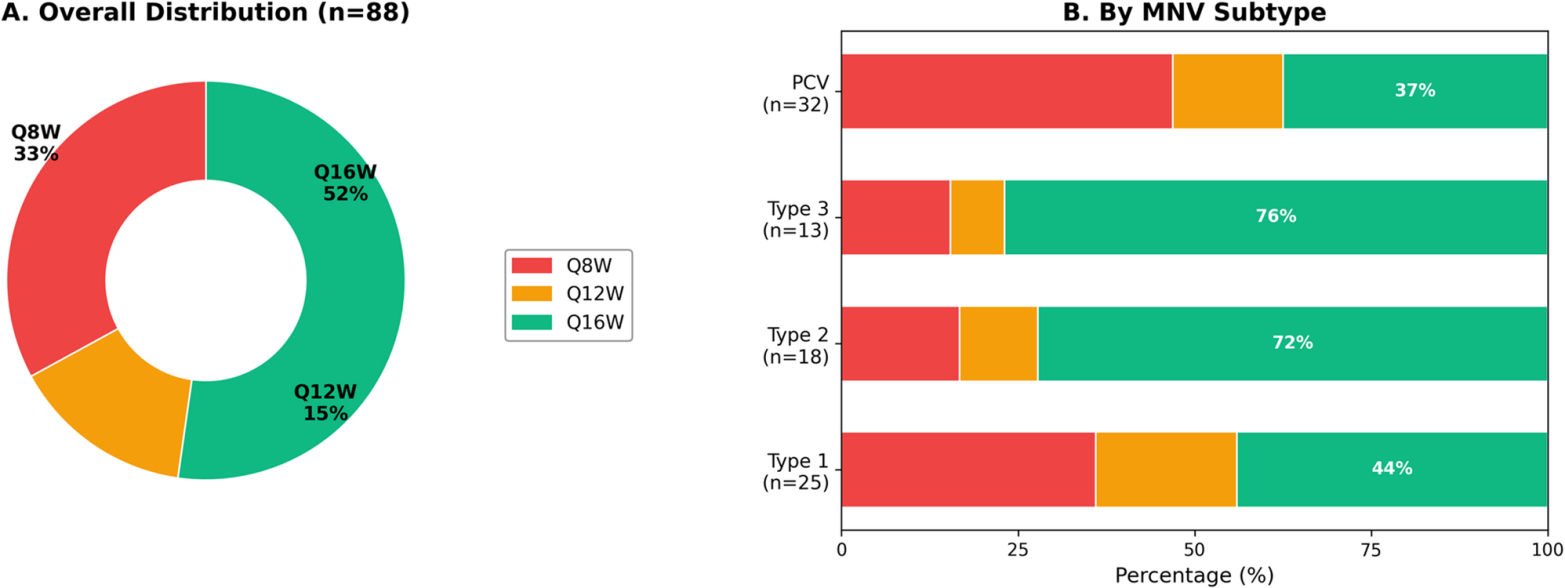
Treatment interval distribution at week 60. (**A**) Overall treatment interval distribution at week 60 (*n* = 88): Q8W 33.0%, Q12W 14.8%, Q16W 52.3%. (**B**) Distribution by MNV subtype showing Q16W achievement rates: type 3 (76.9%), type 2 (72%), type 1 (44%), and PCV (37.5%)

Because intervals were assigned once at weeks 20/24 and maintained thereafter without shortening, the week 60 distribution reflects the interval assigned immediately after the loading phase: Q8W 33.0% (*n* = 29), Q12W 14.8% (*n* = 13), and Q16W 52.3% (*n* = 46). Overall, 67.0% achieved ≥Q12W intervals. Q16W achievement rates varied significantly by MNV subtype: type 3 76.9%, type 2 72.2%, type 1 44.0%, and PCV 37.5% (χ²(3) = 9.52, *P* = 0.023; Fig. 4).

### Safety

Among 168 eyes in the safety analysis, there was 1 case of mild vitritis and 2 cases of RPE tear, with no cases of endophthalmitis or retinal vasculitis reported. The vitritis case was managed by discontinuation of faricimab and observation; the intraocular inflammation resolved without further intervention.

## Discussion

This study provides real-world evidence demonstrating faricimab efficacy in treatment-naïve Asian nAMD patients using a treatment regimen adapted from the pivotal trials. The visual acuity gain of + 7.1 letters at week 20 was comparable to the Asian country subgroup analysis by Takahashi et al. (+ 7.1 letters with faricimab) and numerically higher than the non-Asian subgroup (+ 6.1 letters) [10]. It should be noted that our treatment regimen, while sharing the core principle of disease-activity-based interval assignment at weeks 20 and 24, did not replicate the more complex and rigorously controlled protocols used in TENAYA and LUCERNE.

The high PCV prevalence (36.4%) in our cohort reflects typical Asian nAMD demographics, consistent with the TENAYA/LUCERNE Asian subgroup characteristics [10]. This demographic similarity, combined with a larger sample size (88 vs. 61 eyes), strengthens the validity of our real-world validation.

Our findings are consistent with emerging global real-world data. The TRUCKEE study, the largest real-world faricimab study to date, reported comparable visual and anatomical improvements in a mixed treatment-naïve and previously treated population [18]. Similar positive outcomes have been reported from Europe and other regions [19, 20]. Leung et al. demonstrated significant improvements even in treatment-resistant cases [21], while our treatment-naïve cohort showed even more robust responses, suggesting that early initiation with faricimab may optimise outcomes.

The comprehensive anatomical analysis revealed significant improvements across multiple parameters. The CST reduction (− 134 μm) is consistent with the superior anatomical control demonstrated with faricimab in the head-to-head analysis by Cheung et al. [11] The PED reduction (− 43% at week 20) aligns with post hoc analyses from TENAYA/LUCERNE demonstrating greater PED reduction with faricimab compared with aflibercept [11, 12]. This is particularly relevant given the high PCV prevalence in Asian populations, where serous PED is a common finding [14, 15]. 

At week 20, IRF was absent in 93% and SRF was absent in 88% of eyes, consistent with faricimab’s dual Ang-2/VEGF-A mechanism [6, 13]. The choroidal thickness reduction (− 12%) provides additional mechanistic insight. Unlike VEGF-only inhibitors, Ang-2 inhibition may contribute to sustained vascular normalization [5, 6]. Notably, SFCT reduction varied significantly by MNV subtype, with PCV eyes demonstrating the greatest reduction (− 43 μm), consistent with their pachychoroid phenotype and potentially higher Ang-2 levels [16, 17]. The Tie2 receptor activation through Ang-2 blockade may stabilize the vasculature and reduce exudation beyond VEGF inhibition alone [7]. 

Notably, our analysis of fluid presence in the central 1 mm versus 6 mm zones revealed distinct patterns between IRF and SRF. IRF resolution occurred simultaneously in both zones; among eyes with IRF-free central 1 mm at week 20, only 1.3% (1/75) had residual IRF in the surrounding 6 mm zone. In contrast, SRF showed a different pattern, with significantly higher SRF presence in the 6 mm zone than in the central 1 mm zone at week 20 (19.5% vs. 12.2%; McNemar *P* = 0.031). This suggests that while IRF resolves uniformly across the macula, SRF clearance may proceed centripetally, with peripheral fluid persisting after central resolution. These observations describe differential spatial patterns of fluid resolution, although their clinical implications for treatment decisions require further investigation [22].

The Q16W achievement rate of 52.3% positions our real-world results between the global TENAYA/LUCERNE pooled result (45.7%) and the Asian country subgroup (59.6%) [8, 10]. The ≥Q12W rate (67.0%) is lower than the Asian subgroup (91.2%), likely reflecting the inherent differences between controlled trial protocols and individualised real-world treatment decisions [23]. Nevertheless, over half of patients achieved Q16W dosing, demonstrating meaningful clinical durability. The Japan subgroup analysis by Mori et al. showed similar trends, with excellent durability in Asian patients [24]. Q16W achievement rates differed significantly by MNV subtype, with type 2 (72.2%) and type 3 (76.9%) demonstrating higher interval extension rates compared to type 1 (44.0%) and PCV (37.5%) (χ²(3) = 9.52, *P* = 0.023). These findings corroborate previous observations: Freund et al. reported that type 3 eyes required fewer injections than type 1 or type 2 lesions in the HARBOR trial [25], the Spanish FRB registry demonstrated that type 1 MNV required more frequent injections [26], and type 2 lesions showed better anti-VEGF response [27]. The Vision Academy consensus recommended that type 2 lesions may extend beyond Q12W and type 3 lesions are highly sensitive to anti-VEGF, whereas type 1 and PCV require more intensive treatment [28, 29]. Our data provide quantitative interval benchmarks that translate these qualitative recommendations into clinical practice for faricimab in Asian patients.

Concerns regarding intraocular inflammation with newer anti-VEGF agents have been raised [30, 31]. In our cohort, faricimab demonstrated a safety profile consistent with established anti-VEGF agents and comparable to the Asian subgroup of pivotal trials [8, 10]. No cases of clinically significant intraocular inflammation, retinal vasculitis, or arterial occlusion were observed.

Limitations include the retrospective design, single-centre setting, and relatively short follow-up duration. The lack of a comparator arm limits direct efficacy comparisons with other anti-VEGF agents. The exclusion of patients who did not meet Korean reimbursement criteria may have introduced selection bias toward better responders. Future prospective studies with longer follow-up will help confirm these findings.

## Conclusions

Real-world outcomes of faricimab in treatment-naïve Asian nAMD patients were comparable to the TENAYA/LUCERNE Asian subgroup results. The visual acuity gain, anatomical improvements including CST, PED, SFCT reduction, and fluid resolution rates closely matched pivotal trial findings. These results support faricimab as an effective first-line treatment option for Asian patients with nAMD in a real-world setting.

## Data Availability

The datasets used during the current study are available from the corresponding author on reasonable request.

## Abbreviations

nAMD: Neovascular age-related macular degeneration
VEGF: Vascular endothelial growth factor
Ang-2: Angiopoietin-2
BCVA: Best-corrected visual acuity
CST: Central subfield thickness
PED: Pigment epithelial detachment
SFCT: Subfoveal choroidal thickness
IRF: Intraretinal fluid
SRF: Subretinal fluid
PCV: Polypoidal choroidal vasculopathy
MNV: Macular neovascularization
SD-OCT: Spectral-domain optical coherence tomography
ETDRS: Early Treatment Diabetic Retinopathy Study
Q4W: Every 4 weeks
Q8W: Every 8 weeks
Q12W: Every 12 weeks
Q16W: Every 16 weeks
IOI: Intraocular inflammation
RPE: Retinal pigment epithelium
